# Declined connectivity of thalamus and dorsomedial prefrontal cortex in post-stroke cognitive impairment delineated by lesion network mapping

**DOI:** 10.1093/braincomms/fcag112

**Published:** 2026-03-28

**Authors:** Ting Liu, Yingjie Tang, Yu Wang, Sisi Jiang, Junxia Chen, Lin Ma, Lijuan Li, Cheng Luo, Qifu Li

**Affiliations:** Department of Neurology, The First Affiliated Hospital of Hainan Medical University, Haikou 570100, Hainan Province, China; Key Laboratory of Brain Science Research & Transformation in Tropical Environment of Hainan Province, Hainan Medical University, Haikou 570100, China; The Clinical Hospital of Chengdu Brain Science Institute, MOE Key Lab for Neuroinformation, Center for Information in Medicine, School of Life Science and Technology, University of Electronic Science and Technology of China, Chengdu 610054, China; The Clinical Hospital of Chengdu Brain Science Institute, MOE Key Lab for Neuroinformation, Center for Information in Medicine, School of Life Science and Technology, University of Electronic Science and Technology of China, Chengdu 610054, China; The Clinical Hospital of Chengdu Brain Science Institute, MOE Key Lab for Neuroinformation, Center for Information in Medicine, School of Life Science and Technology, University of Electronic Science and Technology of China, Chengdu 610054, China; The Clinical Hospital of Chengdu Brain Science Institute, MOE Key Lab for Neuroinformation, Center for Information in Medicine, School of Life Science and Technology, University of Electronic Science and Technology of China, Chengdu 610054, China; Department of Neurology, The First Affiliated Hospital of Hainan Medical University, Haikou 570100, Hainan Province, China; Key Laboratory of Brain Science Research & Transformation in Tropical Environment of Hainan Province, Hainan Medical University, Haikou 570100, China; Department of Neurology, The First Affiliated Hospital of Hainan Medical University, Haikou 570100, Hainan Province, China; Key Laboratory of Brain Science Research & Transformation in Tropical Environment of Hainan Province, Hainan Medical University, Haikou 570100, China; Department of Neurology, The First Affiliated Hospital of Hainan Medical University, Haikou 570100, Hainan Province, China; The Clinical Hospital of Chengdu Brain Science Institute, MOE Key Lab for Neuroinformation, Center for Information in Medicine, School of Life Science and Technology, University of Electronic Science and Technology of China, Chengdu 610054, China; Research Unit of NeuroInformation, Chinese Academy of Medical Sciences, Chengdu 2019RU035, P. R. China; Department of Neurology, The First Affiliated Hospital of Hainan Medical University, Haikou 570100, Hainan Province, China; Key Laboratory of Brain Science Research & Transformation in Tropical Environment of Hainan Province, Hainan Medical University, Haikou 570100, China

**Keywords:** post-stroke cognitive impairment, lesion network mapping, thalamus, memory, dorsomedial prefrontal cortex

## Abstract

Post-stroke cognitive impairment (PSCI) is a common complication of stroke. Stroke events can often cause functional changes in distant brain regions beyond the lesion, which may be the underlying cause of PSCI. However, there are still no consistent imaging features with specificity for PSCI. In the present study, we utilized lesion network mapping analysis to address this question. The left thalamus was the only lesion of interest that we converged on, and it mapped the cognitive impairment circuit of PSCI. Patients with severe cognitive impairment had a higher proportion of lesions falling into the cognitive impairment circuit than those with mild cognitive impairment. From the perspectives of brain function, neurotransmitters and molecular genetics, we found that spatial regions of the circuit are strongly associated with memory-related functions. Interestingly, the cognitive impairment circuit from PSCI does share high spatial similarity with the published memory circuit. Besides, reduced functional connectivity in the left thalamus and left dorsomedial prefrontal cortex may be a crucial reason for cognitive decline in PSCI. Overall, our study identifies a cognitive impairment circuit in PSCI, which impairment may be an important mechanism for memory disorders in PSCI. Connectivity of the left thalamus and dorsomedial prefrontal cortex is expected to serve as a biomarker and new target of neuromodulation therapy.

## Teaser

Liu *et al*. pointed out that the lesions of patients with cognitive impairment after acute mild stroke map to a common network. The connectivity of the left thalamus and the dorsomedial prefrontal cortex may be a crucial reason for cognitive decline in post-stroke cognitive impairment.

## Graphical Abstract

**Graphical Abstract:**
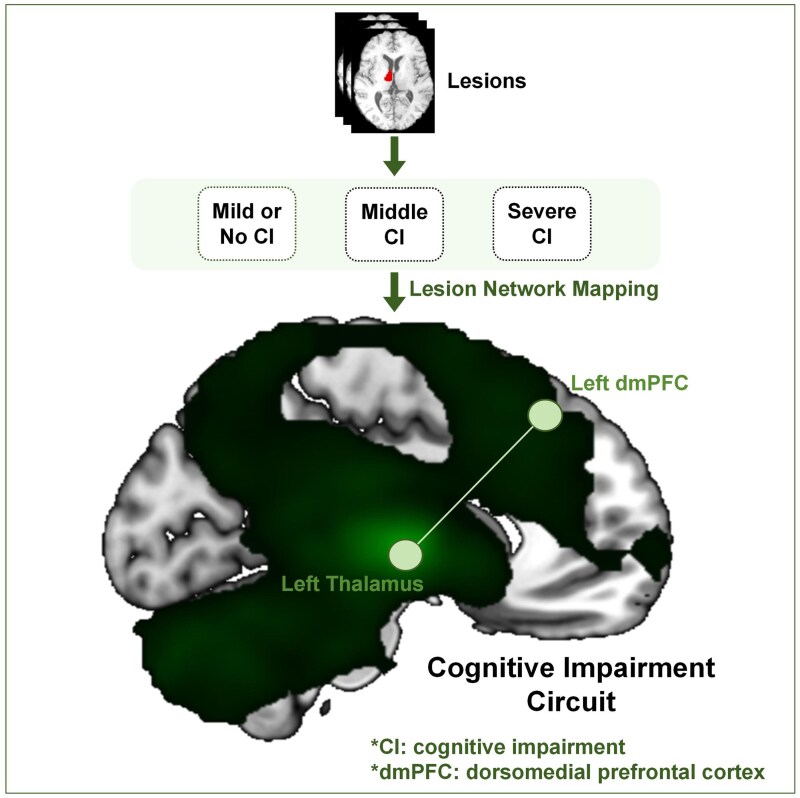

## Introduction

Post-stroke cognitive impairment (PSCI) is a common complication caused by stroke that greatly affects patients’ work and life and is associated with stroke recurrence.^1^ Approximately 44% of patients develop global cognitive impairment 2–6 months after stroke.^2^ However, deficits in cognitive function have been clinically observed early in the onset of stroke, and patients often have impairment in one or more cognitive domains. Despite the fact that this complication has become very common in clinical practice, no specific cognitive scale has been developed for PSCI. For this reason, the Mini-Mental State Examination (MMSE) and the Montreal Cognitive Assessment (MoCA) are the most prevalent tools used in clinical and scientific research to assess the cognitive level of stroke patients.^3^ However, there is often heterogeneity in the scores of the same subject on both scales in cognitive assessment. It is difficult to consider the use of both scales simultaneously in studies to classify the degree of cognitive impairment.

Stroke events can often cause functional changes in distant brain regions beyond the lesion that are at the network level.^4^ Even a smaller infarction can cause diminished attention and reduced processing of information, causing widespread disruption of cognitive networks.^4-6^ Even studies on the Default Mode Network (DMN) have shown that stroke-induced damage to any of the localized brain region structures may lead to abnormal functioning of the DMN.^7^ Stroke affects remote connections between the cerebral hemispheres, which may indirectly influence other brain networks.^8^ Nonetheless, the PSCI common network with specificity is currently unavailable. In recent years, network mapping analysis seems to be the most promising analytical approach to solve this problem. Since disease-induced connectional diaschisis has occurred in patients, studies in patients are not conducive to mapping the full picture of disease-related or symptom-related networks.^9^ Thus, networks/circuits based on the location of injured brain regions mapped in healthy connectivity are proposed.^10^ The traditional Lesion network Mapping adopts the Voxel-based Lesion Symptom Mapping method.^11^ In order to better identify the relationship between lesions and symptoms, the support vector regression lesion symptom mapping method based on machine learning has been developed and applied.^12,13^ However, this is not suitable for symptom-specific network recognition under small sample size data, especially the differences in network under different severity levels of symptoms. Structural mapping network analysis of lesions revealed that cognitive performance in stroke patients was related to the degree of structural disconnection.^14^ While functional network mapping analysis of lesions is less common in PSCI, there have been no studies of lesion mapping networks according to different levels of cognitive impairment after stroke.

**Figure 1:**
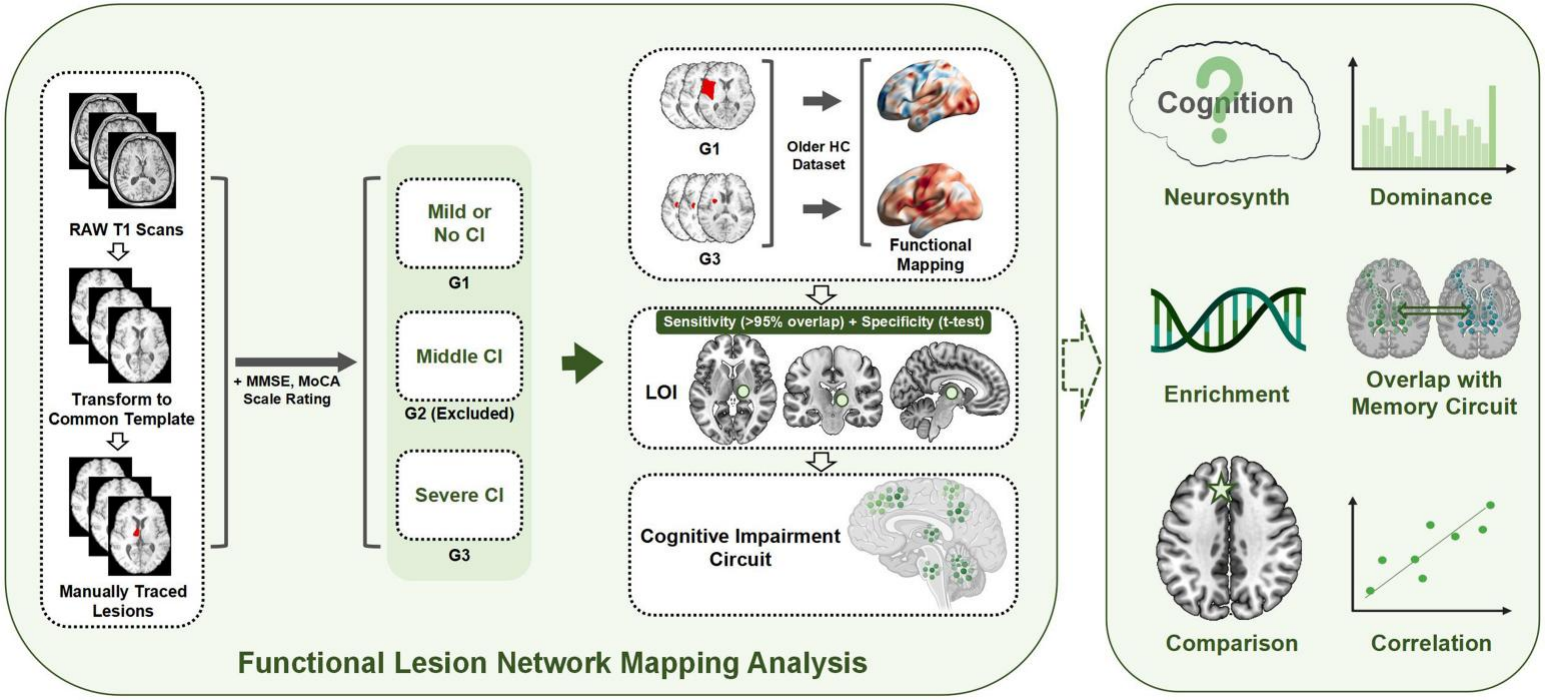
**Flowchart of the analysis.** MMSE: Mini-Mental State Examination; MoCA: Montreal Cognitive Assessment; CI: Cognitive Impairment; HC: Healthy Control; LOI: Lesion of Interest. Created in BioRender. Liu, T. (2026) https://BioRender.com/ouvftyf.

Therefore, we intend to use lesion network mapping to explore lesions of interest for PSCI and mapped circuit based on different cognitive performances in patients with first-onset mild stroke. Characterising the cognitive impairment circuit of PSCI at the level of brain function, neurotransmitters and molecular genetics. Finally, we observed differences in the circuit and its relationship with cognitive scores in patients with PSCI, aiming to inform clinical biomarkers of and targets for neuromodulatory treatments for PSCI. The detailed flowchart is shown in Fig. 1.

## Materials and methods

This study was conducted in accordance with the Declaration of Helsinki and was approved by the Ethics Committee of the First Affiliated Hospital of Hainan Medical University. All participants signed informed consent.

### Subjects

Inclusion criteria for stroke patients: (1) Patients with first acute ischaemic stroke within 2–7 days of onset, age ≥ 40 years; (2) National Institute of Health stroke scale (NIHSS) score ≤ 5. The diagnostic criteria for cerebral infarction were in accordance with the Chinese 2018 Guidelines for the Diagnosis and Treatment of Acute Ischaemic Stroke in China; (3) No severe aphasia and the ability to cooperate in completing neuropsychological assessment; (4) No signs of cognitive decline prior to the onset of the disease as expressed by the patients and their families; (5) Stable vital signs and no progression of the disease. Exclusion criteria: (1) Non-first stroke; (2) Patients with severe clinical diseases, such as heart failure, respiratory failure, renal failure and cancer; (3) Patients with intracranial tumours, traumatic brain injury and risk of seizures; (4) Contraindications to MRI scanning, such as metallic foreign bodies in the body, claustrophobia and so on.

In this study, 77 patients with acute mild ischaemic stroke in the First Affiliated Hospital of Hainan Medical University were collected, and data collection included clinical information, magnetic resonance and neuropsychological assessment. All assessments were completed by four specialized neurologists on the enrolled patients within 3 days of admission. Neuropsychological assessments included MMSE, MoCA, Hamilton Depression Scale (HAMD) and Hamilton Anxiety Scale (HAMA), encompassing temporal and spatial orientation, memory, attention, language and executive ability. After rigorous screening, 16 cases with incomplete data, one case with an NIHSS score > 5 and five cases with poor image data were excluded. Finally, 55 patients with acute mild ischaemic stroke were included in this study.

In order to avoid the effects of bias associated with selecting a particular cognitive scale in isolation, we used the MOCA and MMSE for grouping. Scale scores are labeled on a 0–3 scale based on symptom severity [MOCA^15^: Normal 26–30 (0 scale), Mild 18–25 (1 scale), Moderate 10–17 (2 scale), Severe 0–9 (3 scale); MMSE^16,17^: Normal 24–30 (0 scale), Mild 18–23 (1 scale), Moderate 10–17 (2 scale), Severe 0–9 (3 scale)], with larger scales indicates that the symptoms are approximately severe. Each participant received two levels, and the sum of the two levels ranged from 0 to 6 for all participants. Participants with scores in the range of 0–1 were categorised into the no or mild cognitive impairment group (G1), those with scores of 2–3 into the moderate cognitive impairment group (G2), and those with scores of 4–6 were categorized into the severe cognitive impairment group (G3).

### MRI acquisition

MRI data were acquired using a 3T magnetic resonance GE Discovery MR750 scanner from the First Affiliated Hospital of Hainan Medical University. During the scanning process, all subjects tried to relax as much as possible, closed their eyes and did not perform any specific thinking activities while remaining awake. T1-weighted structural images were acquired with the T1-3DFSPGR sequence with the following parameters: repetition time (TR): 6.012 ms, echo time (TE): 1.968 ms, flip angle (FA): 9°, matrix: 256 × 256, field of view (FOV): 25.6 cm × 25.6 cm, layer thickness: 1 mm, voxel size: 1 × 1 × 1 mm^3^, and 152 consecutive layers were scanned in axial position. Functional magnetic resonance image acquisition was performed with an echo planar imaging sequence with the following parameters: TR: 2000 ms, TE: 30 ms, FA: 90°, FOV: 24 cm × 24 cm, matrix: 64 × 64, layer thickness: 4 mm, gap: 0.4 mm, voxel size: 3.75 × 3.75 × 4 mm^3^, and 255 whole brain images were acquired each time.

### MRI data preprocessing

In this study, we processed the resting-state data using SPM12 (https://www.fil.ion.ucl.ac.uk/spm/) and DPABI (https://rfmri.org/DPABI) toolboxes along with custom MATLAB scripts. The high-resolution T1-weighted magnetic resonance images were processed first. Initially, we checked all images for artefacts and reoriented the images, aligning the origin to the anterior commissure. Subsequently, we performed neck removal and skull stripping, and applied DARTEL to segment the T1 images, addressing intensity inhomogeneity through tissue segmentation and bias correction.

The preprocessing pipeline for fMRI data consisted of the following steps: (1) Converting all functional images to an NIfTI-compatible format and discarding the initial five volumes (10 s) to mitigate the effects of scanner equilibration; (2) Applying slice timing correction to adjust for differences in acquisition times across slices within each volume; (3) Correcting head motion by realigning the images; (4) Similarly, converting the T1 structural images to NIfTI-compatible format, and coregistering the processed T1 structural images to the functional image space; (5) Using DARTEL for spatial normalisation and resampling voxel size to 3 × 3 × 3 mm^3^; (6) Applying spatial smoothing with an 8 mm full-width at half-maximum Gaussian kernel^18^ to improve signal-to-noise ratio and accommodate anatomical variability; (7) Performing regression analysis to remove the effects of Friston 24 head motion parameters, as well as signals from white matter and cerebrospinal fluid.

### Lesion segmentation

Each patient had a focal brain lesion visible on structural MRI images with clear boundaries. Initially normalized to the Montreal Neurological Institute (MNI) 152 template space, lesions were manually delineated in the standard space by a neurologist unaware of cognitive ratings, and each lesion's anatomical accuracy was reviewed by a neurologist. The segmented lesions were binarized, assigning a value of 1 to voxels within the lesion and 0 to all other voxels.

### Lesion network mapping

Lesion network mapping was utilized to investigate the resting-state functional connectivity between lesioned regions and all other voxels in the brain. Specifically, for each stroke patient's lesioned region, Pearson correlation coefficients between the lesioned regions and all other voxels in the brain were computed using resting-state data from 91 older healthy controls (HC); the resulting map represents the lesion network corresponding to each lesion.

Sensitivity analysis was conducted for all stroke patients, where one-sample *t*-tests were performed on each lesion network mapping from the 91 older HC. The resulting *t* maps were binarized and overlaid, identifying regions with an overlap exceeding 95% as sensitivity maps (threshold: *t* > 7, voxel-wise Family-Wise Error (FWE) correction, *P* < 10^−6).

Specificity was evaluated by comparing the lesion mapping network between patients with severe cognitive impairment (G3) and those with mild or no impairment (G1).^19^ Voxelwise two-sample *t* test were performed on the lesion mapping network of the two groups, employing FWE correction for multiple comparisons (*P* < 0.05) to mitigate the influence of other factors, such as regions vulnerable to stroke.

To identify the lesion of interest (LOI), we selected overlapping regions between the sensitivity and specificity maps. Functional connectivity between the LOI and all brain voxels was computed using resting-state data from 91 older HC. One-sample *t* test were performed (threshold: *t* > 5, FWE correction, *P* < 10^−6) to establish the cognitive impairment circuit (see Supplementary Method 1).

### Meta-analytical decoding

We explored the cognitive correlations of the cognitive impairment circuit through Meta-Analytical Decoding. We utilized the Neurosynth database (https://neurosynth.org/decode/) for decoding functional associations with brain regions. Neurosynth is a web platform designed for Meta-analysis in fMRI research, facilitating rapid identification of cognitive functions associated with specific brain regions. Using the Neurosynth brain region correlation method in Python 3.12 with NiMARE v0.2.2 toolkit, we linked the binarized circuit data with term weights across all themes in the Neurosynth dataset. Anatomical terms were excluded, and the resulting correlations were visualized as word clouds, where the size of each term reflects its strength of association.

### Dominance analysis

Dominance analysis, a method developed by Budescu *et al*., was employed to determine the relative importance of predictor variables in multivariate regression equations.^20^ Unlike other methods assessing predictor variable importance, dominance analysis considers both the direct effects of predictor variables and their interactions, providing strong interpretability. In our study, the cognitive impairment circuit was used as the criterion variable Y, while 18 different neurotransmitters^21^ (5-HT1A, 5-HT1B, 5-HT2A, 5-HT4, 5-HT6, 5-HT, CB1, D1, D2, DAT, GABAA, H3, M1, mGluR5, MOR, NET, VAChT, and α4β2) served as predictor variables X.

The key to dominance analysis is examining the change in R^2^ (the ‘Dominance’ of each predictor variable in contributing to the overall fit) when each predictor variable is added to submodels not containing that variable (with P predictor variables, there are 2^P^-1 submodels). Total dominance is defined as the mean of ΔR^2^ when adding each individual predictor variable to the 2^P^-1 submodels. The total dominance of all predictor variables equals the R^2^ of the full model. The relative importance of each predictor variable is measured by calculating the proportion of each predictor variable's total average contribution to the R^2^ of the full model, reflecting each variable's contribution to the model.

To assess the model's significance while keeping X variables constant, we randomly permuted the order of Y values to construct the full model in dominance analysis, obtaining the adjusted R-squared. This process was repeated 10 000 times to build a null distribution model, verifying the reliability of the dominance analysis results.

### Gene expression data processing

The gene expression data were sourced from the Allen Human Brain Atlas (AHBA, http://human.brain-map.org/), comprising expression data for 20 737 genes represented by 58 692 probes. This dataset includes samples from one female and five male donors aged between 24 and 57 years old, with brain tissue samples from the right hemisphere available only for two donors. Therefore, we utilized gene expression data exclusively from the left hemisphere. Following recommended procedures, we conducted gene annotation, data filtering, probe selection, sample allocation, gene filtering and spatial effect removal. Using the Human Brainnetome Atlas, we parcelled the left hemisphere gene expression data into 123 regions. The code for gene expression analysis can be found at https://github.com/BMHLab/AHBAprocessing.

### Transcription-neuroimaging association analysis

Due to focusing exclusively on gene data within the left hemisphere cognitive impairment circuit, we derived a standardized gene expression matrix of 112 brain regions by 10 027 genes. To investigate the role of genes associated with the cognitive impairment circuit, we employed Partial Least Squares Regression (PLSR) analysis. In the PLSR model, the gene expression matrix (112 × 10 027) served as the predictor variables, while the *t*-value matrix of the cognitive impairment circuit (112 × 1) served as the response variable. The first component in PLSR is considered most representative of gene expression associated with the cognitive impairment circuit. Bootstrap resampling was utilized to determine the corrected weights for each gene in the first PLSR component, dividing weights by estimated standard errors to obtain corrected Z-scores. PLSR results were validated via 1000 non-parametric permutation tests to assess whether the variance explained by the first component significantly exceeded random expectation. Subsequent analysis involved ranking Z-scores using *t* test and extracting significant [false discovery rate (FDR) corrected, *P* < 0.05] top—ranked genes, including both positive and negative tails.

### Enrichment analysis

In PLS1 of the cognitive impairment circuit, we selected the top 1500 positively associated genes and the top 1500 negatively associated genes in the gene list. Enrichment analysis was performed using Metascape (http://metascape.org/) in the Kyoto Encyclopedia of Genes and Genomes (KEGG), Gene Ontology (GO) and Disease Gene Network (DisGeNET) databases (FDR corrected, *P* < 0.05).

### Comparison of the cognitive impairment circuit and the memory circuit

Previous studies defined an episodic memory circuit using functional connectivity with the subiculum-retrosplenial continuum.^22^ They provided two MNI coordinates for the subiculum-retrosplenial continuum: (−6, −41, 3) and (8, −39, 3), around which 3 mm-radius spherical seed points were generated. Similarly, using these coordinates, we defined a memory circuit akin to the cognitive impairment circuit. Finally, we calculated the overlap between the cognitive impairment circuit and the episodic memory circuit.

### Comparison of different CI groups

To further elucidate whether there are differences in cognitive impairment circuits following stroke of varying severity, we compared the cognitive impairment circuits between the G1 and G3 groups. Specifically, we conducted additional preprocessing on resting-state fMRI data of both patient groups, where signals from lesion areas were masked by signals from the contralateral hemisphere. We computed functional connectivity (FC) at the voxel level between LOI and the cognitive impairment circuits. Subsequently, a two-sample *t-*test was applied to FC values (corrected using Gaussian Random Field (GRF) at voxel level *P* < 0.01, cluster level *P* < 0.05) to identify significant differences. Peak coordinates of regions showing significant differences were selected as centres to define spherical regions with a 3 mm radius. Average FC values within these regions were calculated. Next, Spearman's correlation was computed between the average FC values and cognition scores.

## Results

### Clinical information and behavioural results

We divided 55 patients with acute mild ischaemic stroke into a mild or no CI group (G1 = 25, Age: 57.4 ± 7.6, 18 Males), a middle CI group (G2 = 15, Age: 63.2 ± 13.8, 12 Males), and a severe CI group (G3 = 15, Age: 63.7 ± 10.0, 12 Males) based on the level of cognitive function. MMSE and MOCA scores and their subscores were statistically significant among the three groups. Triglycerides were significantly different between G2 and G3. Except for triglycerides, no other risk factors showed significant differences between the groups. Other detailed results are presented in Table 1.

**Table 1:** Clinical information and behavioural results

	Mild/No CI (G1, *N* = 25)	Middle CI (G2, *N* = 15)	Severe CI (G3, *N* = 15)	*P* value (*F*/*X*^2^/H)
Gender (M/F)	18/7	12/3	12/3	0.785
Age (Year)	57.4 ± 7.6	63.2 ± 13.8	63.7 ± 10.0	0.097
Education (Year)	12.0 (8.5, 12.0)	9.0 (6.0, 12.0)	9.0 (6.0, 12.0)	0.149
Smoke (%)	12 (48%)	5 (34%)	7 (47%)	0.639
Drunk (%)	8 (32%)	5 (34%)	5 (34%)	0.995
HBP (%)	16 (64%)	7 (47%)	7 (47%)	0.438
Diabetes (%)	8 (32%)	6 (40%)	3 (20%)	0.489
CAD (%)	1 (4%)	0 (0%)	0 (0%)	0.543
NIHSS	2.1 ± 1.7	2.5 ± 1.4	1.9 ± 1.4	0.554
MoCA (Total)	22.0 (20.5, 25.5)	16.0 (15.0, 18.0)	9.0 (6.0, 12.0)	**<0.001^abc^**
Execution	4.0 (3.0, 4.0)	2.0 (0, 3.0)	0 (0,1.0)	**<0.001^abc^**
Name ＆ Language	4.0 (3.5, 5.0)	3.0 (2.0, 4.0)	2.0 (0, 3.0)	**<0.001^bc^**
Attention ＆ Calculation	5.0 (5.0, 6.0)	4.0 (3.0, 5.0)	2.0 (1.0, 3.0)	**<0.001^abc^**
Memory	2.0 (1.0, 4.0)	1.0 (0, 2.0)	0 (0, 0)	**<0.001^bc^**
Orientation	6.0 (5.0, 6.0)	5.0 (5.0, 5.0)	2.0 (1.0, 4.0)	**<0.001^abc^**
MMSE (Total)	26.7 ± 1.5	21.3 ± 2.7	11.2 ± 4.8	**<0.001^abc^**
Orientation	9.0 (8.0, 9.0)	6.0 (6.0, 8.0)	3.0 (1.0, 5.0)	**<0.001^abc^**
Attention ＆ Calculation	5.0 (4.0, 5.0)	3.0 (1.0, 5.0)	0 (0.0, 2.0)	**<0.001^abc^**
Memory	6.0 (5.0, 6.0)	4.0 (4.0, 5.0)	3.0 (2.0, 4.0)	**<0.001^ab^**
Language	3.0 (2.0, 3.0)	3.0 (2.0, 3.0)	2.0 (1.0, 2.0)	**<0.001^bc^**
Execution	5.0 (5.0, 6.0)	6.0 (3.0, 5.0)	2.0 (1.0, 3.0)	**<0.001^bc^**
HAMA	5.0 (4.0, 8.5)	8.0 (5.0, 11.0)	8.0 (6.0, 11.0)	0.272
HAMD	8.0 ± 5.0	9.7 ± 5.1	9.5 ± 5.7	0.500
TG	2.2 (1.4, 4.0)	4.8 (2.6, 5.7)	1.7 (0.9, 2.6)	**0.034^c^**
LDL	3.3 ± 0.8	3.3 ± 0.6	3.3 ± 0.9	0.989
HCY	11.3 (9.3, 18.4)	11.9 (10.4, 16.7)	9.6 (8.2, 13.6)	0.175
HbALc	6.0 (5.6, 9.8)	6.1 (5.6, 10.3)	6.2 (5.9, 8.9)	0.892
Infarction Side (L/R/B)	11/12/2	7/8/0	8/6/1	0.806
Lesion Location				
Frontal Lobe	0 (0%)	2 (11%)	1 (5%)	-
Temporal Lobe	0 (0%)	1 (5%)	2 (11%)	-
Parietal Lobe	1 (4%)	3 (17%)	0 (0%)	-
Occipital Lobe	1 (4%)	1 (5%)	2 (11%)	-
Insula Lobe	0 (0%)	2 (11%)	1 (5%)	-
Subcortical Regions	18 (69%)	6 (33%)	9 (50%)	-
Brainstem	3 (12%)	3 (17%)	3 (17%)	-
Cerebellum	3 (12%)	0 (0%)	0 (0%)	-
Lesion Volume (*n*, voxel 3 × 3 × 3mm^3^)	39.0 (25.0, 56.0)	44.0 (31.0, 62.0)	68.0 (29.0, 187.0)	0.159

Note: CI: Cognitive Impairment; *N*: Number; M: Male; F: Female; HBP: High Blood Pressure; CAD: Coronary Artery Disease; NIHSS: National Institute of Health Stroke Scale; MoCA: Montreal Cognitive Assessment; MMSE: Mini-Mental State Examination; HAMA: Hamilton Anxiety Scale; HAMD: Hamilton Depression Scale; TG: Triglyceride; LDL: Low Density Lipoprotein; HCY: Homocysteine; HbALc: Glycated Hemoglobin; L: Left; R: Right; B: Bilateral. ‘a’ represents the comparison of G1 and G2, *P* < 0.05; ‘b’ represents the comparison of G1 and G3, *P* < 0.05; ‘c’ represents the comparison of G2 and G3, *P* < 0.05.

### Localisation of the lesion associated with cognitive impairment

**Figure 2:**
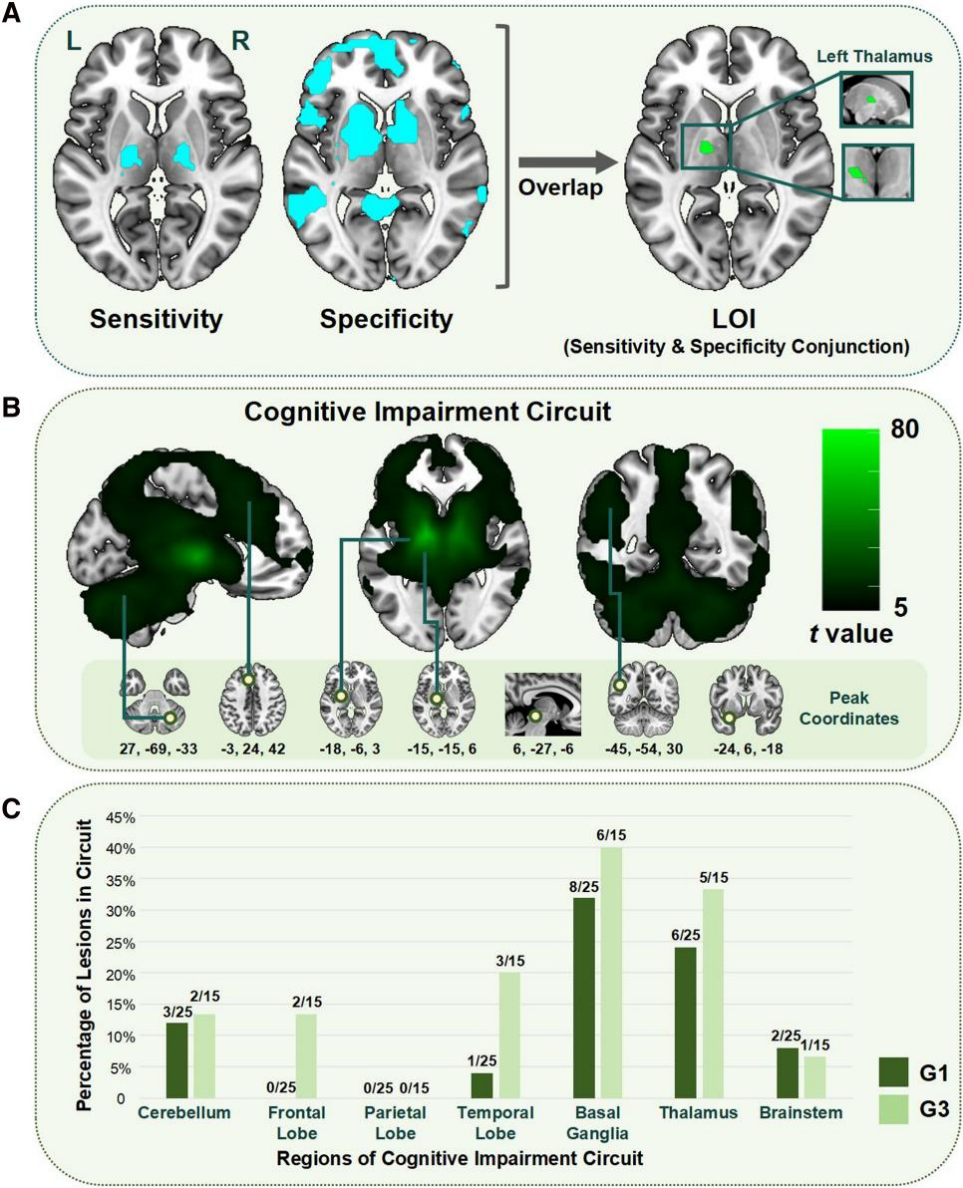
**Lesion of interest and cognitive impairment circuit.** (**A**) indicates the localisation of lesion associated with cognitive impairment; (**B**) indicates the cognitive impairment circuit, and the peak coordinates of the different regions, including cerebellum Crus1, left medial superior frontal gyrus, left superior temporal pole, left pallidum, right midbrain, left angular gyrus and left thalamus; (**C**) indicates the proportion of G1 and G3 lesions falling in different regions of the circuit (G1: *n* = 25, G3: *n* = 15). L: Left; R: Right; LOI: Lesion of Interest.

We found that the lesions in G1, G2 and G3 had a similar range of distribution over brain regions, with the subcortical regions having the highest number of lesions in each subgroup, as shown in Table 1. In order to make our results more accurate, the G2 group was excluded from the subsequent analysis. Considering that most of the stroke subjects were elderly, we selected 91 older HC without cognitive impairment, aged 66.77 ± 7.32 years. By sensitivity and specificity analyses, we identified the LOI associated with the onset of cognitive impairment as the left thalamus, as shown in Fig. 2A Also, as a supplementary analysis, After characterising the lesion on the original anatomical image, we registered the anatomical image to the standard template and found that the left thalamus, as the key LOI, was retained, as shown in Supplementary Method 2 and Supplementary Fig. 1.

### Recognising cognitive impairment circuit

Next, the Cognitive impairment circuit was inscribed on the older HC. The circuit was divided into several major regions (cerebellum, frontal lobe, temporal lobe, basal ganglia, brainstem, parietal lobe and thalamus). We obtained seven peak coordinates including right cerebellum Crus1 (27, −69, −33), left medial superior frontal gyrus (−3, 24, 42), left superior temporal pole (−24, 6, −18), left pallidum (−18, −6, 3), right midbrain (6, −27, −6), left angular gyrus (−45, −54, 30) and left thalamus (−15, −15, 6), as shown in Fig. 2B. To explain the relationship between lesions and cognitive impairment circuit, we found that a higher proportion of lesions fell into the circuit in G3 than in G1 in the cerebellum, frontal lobe, temporal lobe, basal ganglia and thalamus, with the exception of the brainstem and parietal lobe, as shown in Fig. 2C.

### Functional characterisation and receptor/transporter profiles of the cognitive impairment circuit

**Figure 3:**
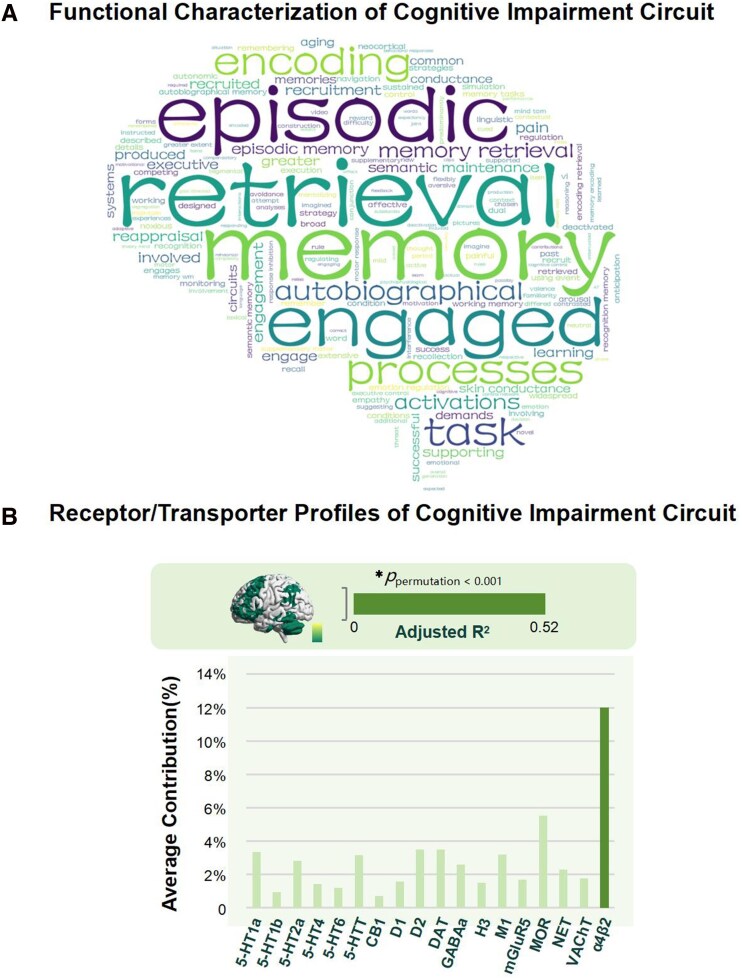
**Functional characterisation and receptor/transporter of cognitive impairment circuit.** (**A**) represents word cloud of top terms related to cognitive impairment circuit using Neurosynth. (**B**) represents the average contribution rate of dominance analysis of 18 neurotransmitter profiles, * represents model is significant, tested 10 000 times by permutation (*P* < 0.001). HT: Serotonin; CB: Cannabinoid; D: Dopamine; DAT: Dopamine Transporter; GABA: Gamma-aminobutyric acid; H: Histamine; M1: M1 acetylcholine receptor; mGluR: Glutamate receptor; MOR: mu-opioid receptor; NET: Norepinephrine transporter; VAChT: VAChT acetylcholine transporter; α4β2: α4β2 acetylcholine receptor.

Although we believe that this circuit is related to the occurrence of cognitive impairment after stroke, further exploration is required to elucidate the specific cognitive functions that this circuit may be involved in. To clarify the possible functional characteristics of this circuit, we utilized Neurosynth to decode the functions associated with brain regions in the cognitive impairment circuit. The results show that the mapped cognitive impairment circuits are associated with ‘retrieval’, ‘memory’, ‘episodic’, ‘engaged’, ‘processes’ and ‘task’ terms, as shown in Fig. 3A.

Based on the publicly available 18 neurotransmitter maps, we are conducting essential explorations of the cognitive impairment circuit at the molecular level. The results of the dominance analysis showed a strong association between the cognitive impairment circuit and α4β2 receptor/transporter density (Adjusted R^2^ value = 0.52; Permutation test 10 000 times, *P* < 0.001). Accordingly, we identified that α4β2 receptor density has the most significant contribution to explaining the cognitive impairment circuit, as shown in Fig. 3B.

### Gene expression profiles of the cognitive impairment circuit

**Figure 4:**
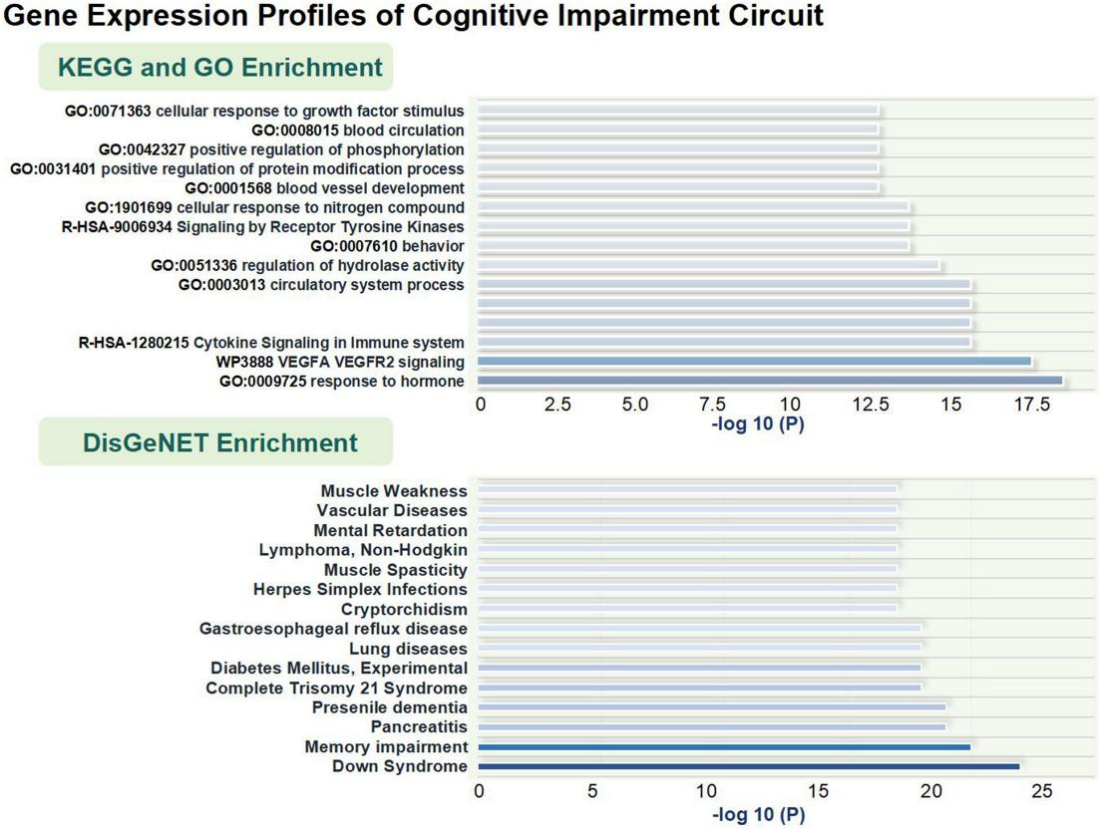
**Gene expression profiles of cognitive impairment circuit (the enrichment analysis of PLS1).** KEGG: Kyoto Encyclopedia of Genes and Genomes; GO: Gene Ontology; DisGeNET: Disease Gene Network.

The underlying mechanisms of the cognitive impairment circuit at the genetic level were explored through cross-sample spatial correlation analyses with permutation tests (1000 times) on the PLS1. Our results demonstrate the top 15 most significant results. KEGG and GO enrichment analyses revealed significant associations of cognitive impairment circuit in biological processes including inflammation, signalling, phosphorus-related regulation, circulation and vascular development, and behaviour, as shown in Fig. 4. DisGeNET enrichment analysis identified multiple significant results related to cognition- and intelligence-related disorders (down syndrome, memory impairment and presenile dementia, etc.), diseases/symptoms of the cerebral vasculature (muscle spasticity, vascular diseases and muscle weakness) and other systemic disorders, as shown in Fig. 4.

### Similarity of cognitive impairment circuit and memory circuit

**Figure 5:**
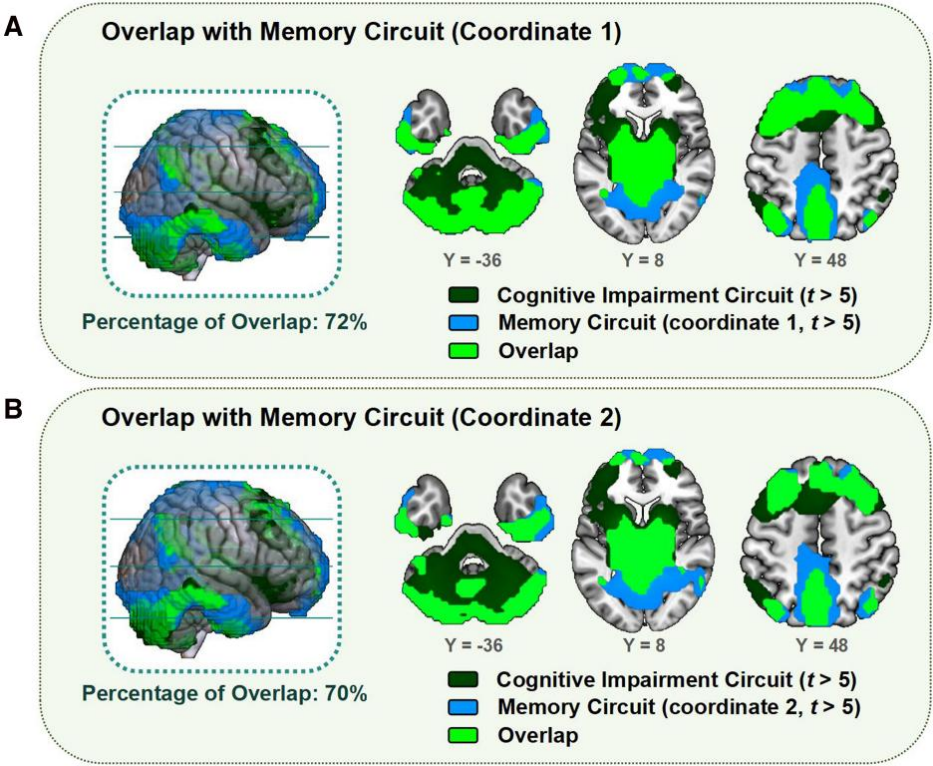
**Overlap of cognitive impairment circuit and memory circuit.** (**A**) Memory circuits are mapped by coordinates 1 (−6, −41, 3) on 91 older HC. (**B**) Memory circuits are mapped by coordinates 2 (8, −39, 3) on 91 older HC.

Functional characterisation and DisGeNET enrichment analysis of the cognitive impairment circuit have revealed its close association with ‘memory’. We use the coordinates of the subiculs-retrosplenial continuum to map the memory circuit. Memory circuit were mapped in older HC based on two coordinates, including ventral and medial prefrontal cortex, thalamus, lateral parietal cortex, lateral temporal cortex and cerebellum. The spatial distribution was similar to that of the cognitive impairment circuit. By calculating the percentage of overlap, the cognitive impairment circuit overlapped 72% with the memory circuit based on coordinate 1, and 70% with the memory circuit based on coordinate 2, as shown in Fig. 5A, B.

### Comparison of mild/no CI and severe CI groups

**Figure 6:**
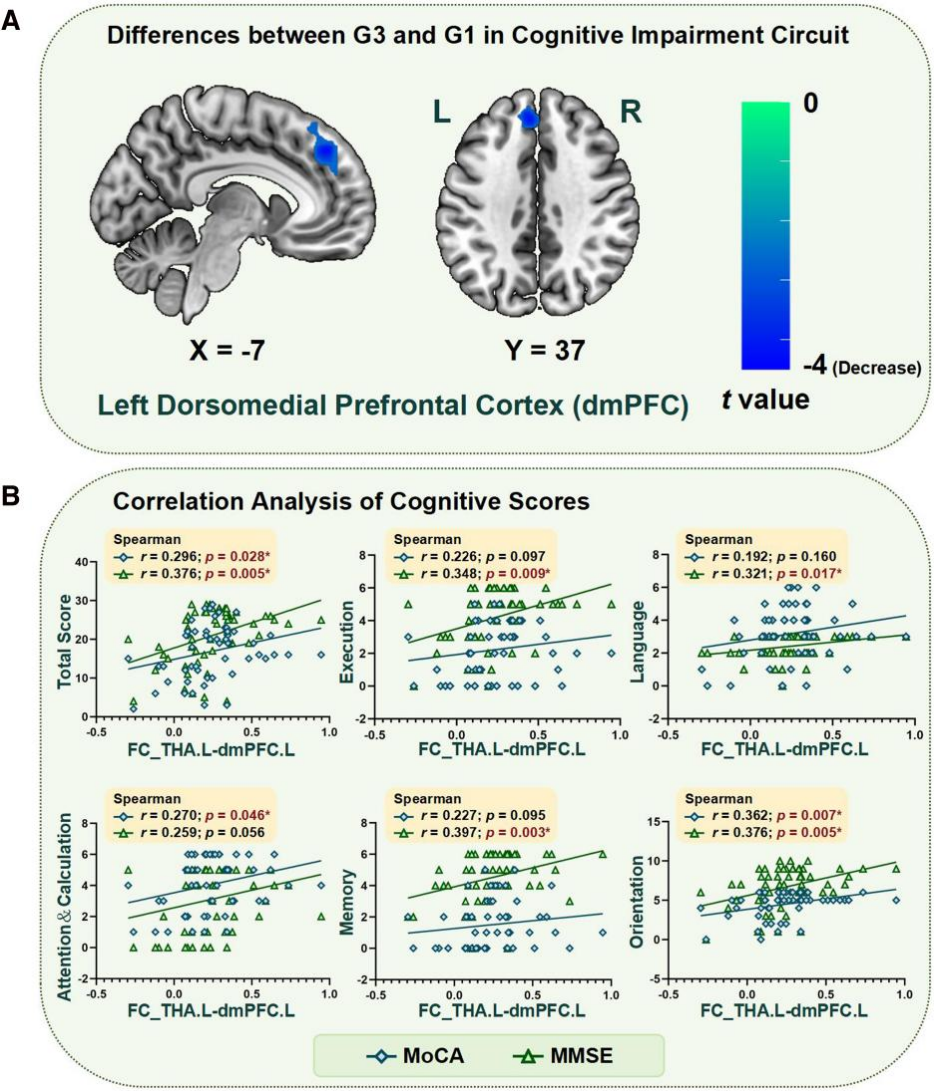
**Comparison of Mild/No CI and severe CI group in cognitive impairment circuit.** (**A**) shows the significant difference between G3 and G1 in the cognitive impairment circuit (two-sample *t* test, *P* < 0.05; GRF correction, two-tailed, voxel level *P* < 0.05, cluster level *P* < 0.05). (**B**) represents the correlation between FC in left thalamus and left dmPFC and cognitive scores (*n* = 55). * represents *P* < 0.05. L: Left; R: Right; FC: Functional Connectivity; THA: Thalamus; dmPFC: Dorsomedial Prefrontal Cortex; MMSE: Mini-Mental State Examination; MoCA: Montreal Cognitive Assessment.

To further clarify whether cognitive impairment circuits differ in various levels of cognitive impairment after stroke, we compared the cognitive impairment circuit in G3 and G1. It was found that G3 had significantly lower FC than G1 in the left dorsomedial prefrontal cortex (dmPFC) (two-sample *t* test, *P* < 0.05; GRF correction, two-tailed, voxel level *P* < 0.01, cluster level *P* < 0.05). What's more, this is consistent with the frontal lobe's peak coordinate, as shown in Fig. 6A. Furthermore, we calculated the correlation between FC in the left thalamus and left dmPFC and cognitive scores, both of which showed significant positive correlations, as shown in Fig. 6B.

Even after controlling the lesion volume, this FC in the left thalamus and left dmPFC still showed a positive correlation trend with each cognitive score, and was significantly positively correlated with the total score of MMSE, as shown in Supplementary Table 1.

## Discussion

In this study, we identified a cognitive impairment circuit in PSCI and found a link between lesions and the circuit. The relationship between circuit and cognition, especially memory, was affirmed from the perspective of functional annotation of brain regions and gene expression. By further analysis, we found that α4β2 receptor density was the most critical among the 18 neurotransmitter profiles for interpreting the cognitive impairment circuit. Interestingly, we found a high spatial similarity between the cognitive impairment circuit and the published memory circuit. In the comparison of severe CI and mild CI, we focused on the left dmPFC. This was consistent with the peak coordinate of the circuit in the frontal lobe and was significantly and positively correlated with the total cognitive scores and their cognitive domain scores. These findings have the potential to be an imaging biomarker for PSCI and provide a new therapeutic target for the neuromodulatory treatment of PSCI.

The majority of those presenting with impairment in more than two cognitive domains during the acute phase of stroke were patients with mild stroke (NIHSS ≤ 5), and impairment of learning and memory was most prevalent.^23^ PSCI is often accompanied by other complications, such as post-stroke depression (PSD). The prevalence of PSD combined with PSCI reached 26.15%.^24^ Patients with first acute mild stroke within 2–7 days of onset were also selected for our study and grouped for severity of cognitive impairment using two scales; anxiety and depression scores did not differ between groups. Likewise, we also compared other risk factors for PSCI, such as education, smoking, diabetes, homocysteine and lipids.^23,25-27^ However, our results showed that TG was significantly higher in G2 than in G3. One study suggested that higher TG levels in the acute phase of ischaemic stroke were associated with PSCI.^28^ To control for all risk factors considered and to derive a cognitive impairment circuit, we excluded G2 in subsequent analyses. The infarction side and lesion location are strongly associated with the development of cognitive impairment. About 25% of strokes occur in the subcortical region, a common cause of vascular dementia.^29,30^ We descriptively counted infarction side and lesion location, and the distribution of lesions in each group was mainly concentrated in the subcortical region.

With the goal of exploring the cognitive impairment circuit, we performed functional mapping analyses on 91 cognitively normal older HC. Actually, lesion-symptom mapping has been applied in a wide range of neuropsychiatric diseases, and it is a well-established method.^31^ It is important to note that essentially all studies used young adults for network mapping. Considering the age of stroke onset, we believe that mapping analyses in healthy individuals need to take into account the potential impact of some natural ageing changes. Typically, in order to explore lesion-induced neurological deficits, voxel-based lesion-symptom mapping is the most commonly used analysis method.^32,33^ However, the problem we sought to address was the different cognitive outcomes resulting from stroke lesions, which required the lesions most associated with exacerbation of cognitive impairment. For the LOI, we identified the left thalamus using sensitivity and specificity analyses. The location of the LOI is biased towards the anterior side of the thalamus, which is usually considered an important part of the neural circuits for learning and memory.^34^

In terms of the distribution of brain regions in the cognitive impairment circuit, the cortical portion appears to have a strong similarity to that of the DMN, executive control network (ECN) and salience network (SN). It is well known that the DMN, ECN and SN comprise the triple-network model of psychopathology, which is involved in cognitive functioning and emotional activity.^35^ Our results suggest that the left anterior thalamus generates a functional mapping to the cortical triple-network model, which is strongly associated with the development of PSCI. In addition, differently, regions of the basal ganglia, thalamus, cerebellum and brainstem in the loop are not in the classical resting-state brain network, and we further supplemented the brain regions associated with the onset of PSCI. The cerebellum has been thoroughly studied from motor control to cognition and emotion.^36,37^ There is evidence that the cerebellum is part of the episodic memory circuit of emotional enhancement, inputting from the anterior cingulate cortex and outputting to the basal ganglia (amygdala/hippocampus).^38^ From the image, our circuit covers most of the area of the cerebellum, which is mapped from the anterior thalamus. The complex role of the cerebellum also includes involvement in executive function, language and spatial information processing.^37^ It is often assumed that brainstem stroke causes cognitive impairment as a relatively rare consequence, thus ignoring the role of the brainstem in the development of cognitive impairment. Brainstem PSCI is commonly characterized by impairments in executive function, working memory and spatial memory capacity.^39^ This may be caused by failure of hierarchical cognitive processing in the fronto-ponto-cerebellar-thalamic circuit.^40^ Taken together, we believe that the involvement of these regions is an important basis for multiple cognitive domain impairments after stroke. Our view is that the cognitive impairment circuit mapped by the LOI provides a better explanation of the disease mechanisms in terms of the distribution of brain regions for the prominent memory impairments, characteristic spatial information processing deficits, and executive function decline in PSCI. Further, we found that the proportion of severe PSCI with lesions located in the circuit is higher, further emphasising the importance of the circuit in explaining the mechanisms of progression of cognitive impairment.

Although looking at the function of each individual brain region, we seem to be able to look for connections to cognitive function. However, this is far from enough to reveal the significance of the whole circuit. We utilized meta-analytical decoding to support our view. Cognitive impairment circuits were found to be associated with memory (episodic and retrieval) and other higher cognitive functions (engaged, processes and tasks). The subiculum-retrosplenial continuum is the hub of the human memory circuit identified by Fox *et al.*, which is most precisely located and associated with memory in the hippocampus.^22^ Animal studies also support the importance of the hippocampus in the memory circuit, with neuro-anatomical pathways suggesting that the processing of memories derives from the dorsal hippocampus, entorhinal cortex, prefrontal cortex, anterior thalamus and the retrosplenial cortex are involved.^41^ Interestingly, the percentage of overlap also demonstrates the high similarity between the cognitive impairment circuit of PSCI and the memory circuit.^22^ Obviously, our LOI (thalamus) is quite different from Michael's LOI (hippocampus). Therefore, we hypothesize that brain network progression in PSCI occurs in a larger circuit similar to the memory circuit, and that the LOI-mapped cognitive impairment circuit reveals features of cognitive deficits and progression of memory deficits in mild stroke.

In recent years, dominance analysis and transcription-neuroimaging association analysis have become a novel approach to explain the molecular mechanisms underlying imaging manifestations being applied in neuropsychiatric disorders.^42-44^ We discovered a significant contribution of the α4β2 receptor in the circuit. The α4β2 receptor is a type of nicotinic ACh receptor (nAChRs) that is highly expressed in the thalamus and is involved in synaptic plasticity in learning, memory and attention.^45,46^ In Alzheimer's disease, a significant reduction in α4β2 receptor utilisation is associated with the development of deficits in situational memory, executive function and attention.^47^ The α4β2 nAChRs agonist ameliorates memory deficits and anxiety-like behaviours in ischaemic mice by suppressing inflammatory factor levels.^48^ The results of the dominance analysis are consistent with our previously advocated viewpoints and complement the molecular mechanisms of the cognitive impairment circuit from a transmitter/receptor perspective, which could support future clinical drug development for the treatment of PSCI. Moreover, it is also necessary to explore the molecular genetic characterisation of the cognitive impairment circuit. Rats with PSCI have a large number of pathological factors, such as expression of inflammatory factors and signalling abnormalities in vivo.^49^ Although we enriched some biological pathways related to inflammation and signalling, we think that there may not be much specificity. However, biological processes such as circulation, vascular development and behaviour apparently provide support from genetics for the cognitive impairment circuit in PSCI, although more research is needed to understand the mechanisms involved. Notably, we also had supporting evidence in a database of disorders. Multiple significantly enriched outcomes were cognition- and intelligence-related disorders, particularly memory disorders. In short, these micro-level transmitter and gene expression profiles are underlying pathological conditions in the cognitive impairment circuit that contribute to the onset and progression of PSCI.

How cognitive impairment circuits can be further applied to the study of clinical diagnosis and mechanisms is a question we should continue to ponder. By comparison, we identified a significant reduction in FC of the left thalamus and left dmPFC in G3 compared to G1. Previous studies have demonstrated that the medial prefrontal cortex is engaged in the processing of memory, functioning together with other brain regions in episodic-like and associative object-recognition memory.^41,50,51^ Ischaemic infarction in the medial prefrontal cortex leads to impairment of spatial and recognition memories.^52^ This is in the direction of our previous hypothesis. This may be an imaging biomarker that distinguishes PSCI from other cognitive impairment disorders. The latest article reviews the brain regions with decreased resting-state functional connectivity in PSCI, including the core brain regions of DMN such as the medial prefrontal cortex and inferior frontal gyrus, which supports the important role of dmPFC in PSCI.^53^ In addition to this, our results suggest that a higher FC of the left thalamus and ipsilateral dmPFC is associated with better cognitive performance. Especially, this is a significant positive correlation with all cognitive domain scores, implying that increasing the FC of the left thalamus and left dmPFC is likely to improve all aspects of cognitive function in stroke patients. There is a lack of research on the neuromodulatory targets of PSCI, and the commonly used target remains the left dorsal lateral prefrontal cortex.^54^ The effectiveness of the rTMS stimulation of dmPFC for the treatment of major depressive disorder and addiction symptoms in schizophrenia has been found.^55,56^ Stimulation of dmPFC improves cognitive function in addition to depression.^57^ Our study provides a new potential candidate for a therapeutic target for PSCI.

There are some limitations. First, the sample size of this study is small compared to other articles of the same type, and the age of older HC is older than that of PSCI. Recently, coordinate-based network mapping analysis has been recognized by researchers.^55^ Our results will be further validated in the future in the coordinates of lesions provided in the published articles. Second, since the cognitive impairment circuit is not a whole-brain map, the dominance analysis and PLS analysis were not subjected to the spin test, and we chose a permutation test to demonstrate their significance. Third, we lack specialized cognitive domain-related scales. Finally, the transcription-neuroimaging association analysis only included gene samples from the left cerebral hemisphere, restricting the generalisability of those findings.

## Conclusion

In conclusion, our study identified a cognitive impairment circuit in PSCI. From different perspectives, we reveal that the spatial regions of the circuit are strongly associated with memory-related functions. Impairment of the circuit may be an important mechanism for memory disorders in PSCI. In addition, we recommend the left thalamus-dmPFC connectivity as a new promising potential biomarker and target for neuromodulation therapy.

## Supplementary Material

fcag112_Supplementary_Data

## Data Availability

Due to privacy and ethical constraints, they are not available to the public. The data for this study are available from the corresponding authors upon reasonable request. The codes for Lesion Network Mapping and Dominance Analysis in this manuscript can be found in the Supplementary methods 3–4. The codes for other methods are clearly stated in the methods section of the manuscript.
