# Supplementary material for: Declined connectivity of thalamus and dorsomedial prefrontal cortex in post-stroke cognitive impairment delineated by lesion network mapping
Source: Brain Commun. 2026 Mar 28;8(2):fcag112. doi: 10.1093/braincomms/fcag112 (PMC13062370; doi:10.1093/braincomms/fcag112)
Supplement: fcag112_Supplementary_Data [file fcag112_supplementary_data.docx]

**Supplementary Method 1. Lesion Network Mapping.**

We focused on seven major brain regions within the circuit: cerebellum, frontal lobe, parietal lobe, temporal lobe, basal ganglia, thalamus, and brainstem. The cortical regions (cerebellum, frontal lobe, parietal lobe) were defined using the Automated Anatomical Labeling (AAL) template developed by Tzourio-Mazoyer et al. in 2002. For subcortical areas like the basal ganglia and thalamus, we utilized the high-precision Tian subcortex parcellation template developed by Ye Tian and colleagues, based on 3T MRI data (<https://github.com/yetianmed/subcortex/blob/master/Group-Parcellation/3T/Subcortex-Only/Tian_Subcortex_S4_3T_label.txt>). The brainstem was analyzed using the Harvard ascending arousal network (AAN) atlases.^1^

**Supplementary Method 2. Lesion-aware normalization pipeline.**

We implemented a lesion-aware normalization pipeline using FSL. For each subject, the T1 image was skull-stripped, the manually drawn lesion mask was binarized, and a subject-specific “healthy mask” (brain mask minus lesion) was used as the FNIRT in-mask so that the lesion does not contribute to the registration cost function. The resulting nonlinear warp was applied to the binary lesion using nearest-neighbour interpolation to preserve binary labels. This approach therefore implements cost-function masking and minimizes registration bias introduced by lesions.^2^

**Supplementary References**

1. Edlow BL, Takahashi E, Wu O, Benner T, Dai G, Bu L, Grant PE, Greer DM, Greenberg SM, Kinney HC, Folkerth RD. Neuroanatomic connectivity of the human ascending arousal system critical to consciousness and its disorders. J Neuropathol Exp Neurol. 2012 Jun;71(6):531-46.
2. Brett M, Leff AP, Rorden C, Ashburner J. Spatial normalization of brain images with focal lesions using cost function masking. Neuroimage. 2001 Aug;14(2):486-500.

**Supplementary Method 3. Code of Lesion Network Mapping (matlab).**

%% =========================================================================

% Lesion 鈫? Gray-matter Functional Connectivity (Lesion-Network Mapping)

%

% Purpose (manuscript-aligned):

% This script computes voxelwise functional connectivity maps for

% lesion seeds. For each lesion (binary mask) or user-defined seed,

% the mean seed time series is correlated with every gray-matter voxel

% in a normative resting-state cohort (used in the manuscript: 91 older HC).

% The subject-level outputs form the basis for lesion-network mapping,

% sensitivity/specificity analyses, and identification of Lesion(s) of

% Interest (LOI) as described in the manuscript.

%

% Outputs (per lesion/seed per subject):

% - FC : Pearson r (ROI 脳 gray-voxel)

% - FZ : Fisher z (atanh of r; numeric safeguards applied)

% - SZ : Within-seed standardized z-score ((r - mean)/std)

% Files are saved as: <subjID>_ROI_Gray_[FC|FZ|SZ].mat

%

% Typical pipeline usage in manuscript:

% 1) Compute lesion 鈫? whole-brain FC maps on the normative HC cohort.

% 2) Run one-sample t-tests across HC lesion-maps to obtain sensitivity maps.

% 3) Compare maps between patient subgroups (specificity) to identify LOI.

% 4) Use overlapping sensitivity & specificity results to define LOI and

% derive the cognitive-impairment circuit (see Methods / Supp. Methods).

%

% Important assumptions & cautions:

% - Lesion/seed masks MUST be in the same space as the functional images.

% The script does NOT perform registration or resampling.

% - Functional data must be preprocessed (realign, coregister, nuisance

% regression, normalization, smoothing, etc.) consistent with manuscript.

% - Correlations are clipped at 卤0.99 before Fisher transform to avoid

% numerical overflow; this mildly attenuates extreme values.

% - Small or poorly defined lesions (few voxels) yield unstable seed time

% series 鈥? treat such outputs with caution in downstream inference.

% - Interpretation of lesion-network results depends on the normative

% connectome (age/scan parameters); mapping performed on age-matched

% controls (as in the manuscript) reduces but does not remove bias.

%

% Implementation notes:

% - Outputs saved in single precision and -v7.3 (HDF5) to support large arrays.

% - Within-seed SZ is computed per ROI across all gray voxels; ROIs with

% zero variance are assigned zeros to preserve matrix shape.

% - Designed to feed directly into group-level statistics (t-tests, GRF/FWE

% corrections) and overlap-based LOI selection described in the paper.

%

% ==========================================================================

clear; clc;

%% --------------------------- Add toolbox paths ---------------------------

% Add required toolboxes (SPM, DPABI, REST) to MATLAB path. Adjust paths

% below to the local installation used for processing and analysis.

addpath(genpath('/home/hujian/sxq/Tang/Tool/DPABI_V6.1_220101'));

addpath(genpath('/home/hujian/sxq/Tang/Tool/spm12'));

addpath(genpath('/home/hujian/sxq/Tang/Tool/REST_V1.8_130615'));

%% ------------------------------ User config -----------------------------

% Directory containing user-defined ROI NIfTI files (.nii). Each ROI is

% expected to be a binary mask (non-zero = seed).

roiFolder = '/home/hujian/TYJ/liuting_new/new_lesion_1016/RE_G1MASK/Reslice_G1MASK';

% Parent folder of subject functional data. Each subject should occupy a

% separate subfolder containing one (or more) .nii functional file(s).

subjectFolder = '/home/hujian/CJX/wangyuSDC_FDC/Data/HC_91/FunImgARWSCF';

% Output root directory. Three subfolders (FC, FZ, SZ) will be created.

outDir = '/home/hujian/TYJ/liuting_new/New_mask_circut_G1_new_1016';

if ~exist(outDir, 'dir'), mkdir(outDir); end

% Subfolders for saved matrices:

outFC = fullfile(outDir, 'FC'); if ~exist(outFC, 'dir'), mkdir(outFC); end

outFZ = fullfile(outDir, 'FZ'); if ~exist(outFZ, 'dir'), mkdir(outFZ); end

outSZ = fullfile(outDir, 'SZ'); if ~exist(outSZ, 'dir'), mkdir(outSZ); end

% Gray-matter mask (NIfTI) in the same space as the functional images.

% This mask defines the target voxels for ROI-to-gray correlations.

grayMatterMaskPath = '/home/hujian/TYJ/liuting_new/GreyMask_02_61x73x61.img';

%% 1. Load all custom ROI NIfTIs and extract voxel indices

% Each ROI is read and its non-zero voxels are stored as linear indices so

% that ROI time series can be computed as the mean across these voxels.

niiList = dir(fullfile(roiFolder, '*.nii'));

nROIs = numel(niiList);

if nROIs == 0

error('No .nii ROI files found in roiFolder. Check path and file extension.');

end

customROI_vox = cell(nROIs,1);

for j = 1:nROIs

[roiData, ~] = y_Read(fullfile(roiFolder, niiList(j).name)); %#ok<NASGU>

mask = roiData > 0;

customROI_vox{j} = find(mask); % linear voxel indices of ROI j

end

%% 2. Enumerate subject directories

% Assumes each subject has a dedicated subfolder under subjectFolder.

subjectDirs = dir(subjectFolder);

subjectDirs = subjectDirs([subjectDirs.isdir] & ~ismember({subjectDirs.name},{'.','..'}));

nSubjects = numel(subjectDirs);

subjNames = cell(nSubjects,1);

if nSubjects == 0

error('No subject subfolders found under subjectFolder. Check organization.');

end

%% 3. Read a sample functional file to determine image dimensions

% This step ensures dimensional consistency between subjects and with the

% gray-matter mask.

sampleSubjFiles = dir(fullfile(subjectFolder, subjectDirs(1).name, '*.nii'));

if isempty(sampleSubjFiles)

error('No .nii file found in example subject folder: %s', subjectDirs(1).name);

end

[sampleVol, ~] = y_Read(fullfile(subjectFolder, subjectDirs(1).name, sampleSubjFiles(1).name));

dims4 = size(sampleVol); % [X Y Z T]

nVox = prod(dims4(1:3));

% Read and validate gray-matter mask dimensions against the sample volume

[grayMaskData, ~] = y_Read(grayMatterMaskPath);

grayMask = grayMaskData > 0;

if ~isequal(size(grayMask), dims4(1:3))

error('Gray-matter mask dimensions do not match functional image spatial dimensions. Resample before proceeding.');

end

grayMatter_vox = find(grayMask);

nGrayVox = numel(grayMatter_vox);

%% 4. Parallel execution setup (Slurm cluster / local parallel pool)

% Progress is reported via a DataQueue (prints a dot per processed ROI).

dq = parallel.pool.DataQueue;

afterEach(dq, @(~)fprintf('.')); % small progress indicator

% Configure cluster settings (customize for local cluster). This block uses

% a Slurm profile; adapt or replace with local parpool(...) if running on

% a non-cluster environment.

c = parcluster('SlurmProfile_SSJ');

c.NumWorkers = 20;

c.SubmitArguments = '-p partCU --nodes=1 --nodelist=cu[08]';

c.ResourceTemplate = '--ntasks-per-node=1 --cpus-per-task=20';

parpool(c, 19, 'IdleTimeout',360);

%% Main parallel loop: compute ROI-to-gray-voxel correlations per subject

% For each subject:

% - load functional data into [nVox x T] (reshaped)

% - extract gray-matter voxel time series [nGrayVox x T]

% - for each ROI compute seed timeseries (mean across ROI voxels)

% - compute Pearson r between ROI seedTS and every gray voxel

% - clip r to [-0.99, 0.99], compute Fisher z (atanh)

% - compute within-ROI standard score (z-score of r across voxels)

% - save three matrices per subject: FC_sub (r), FZ_sub (fisher z), SZ_sub (within-ROI z)

parfor si = 1:nSubjects

subjName = subjectDirs(si).name;

subjNames{si} = subjName;

try

% Locate the subject's .nii file(s). This implementation assumes the

% main 4D time series is present in a single .nii file.

nii = dir(fullfile(subjectFolder, subjName, '*.nii'));

if isempty(nii)

warning('Subject %s: no .nii file found, skipping.', subjName);

continue;

end

[vol, ~] = y_Read(fullfile(subjectFolder, subjName, nii(1).name));

[sx,sy,sz,st] = size(vol);

if prod([sx,sy,sz]) ~= nVox

error('Subject %s: voxel count does not match sample dimensions.', subjName);

end

% Reshape to [nVox x T] and select gray-matter voxels -> [nGrayVox x T]

VxT = reshape(vol, [], st); % [nVox x T]

grayVxT = VxT(grayMatter_vox, :); % [nGrayVox x T]

% Preallocate subject-level matrices (single precision to save memory)

FC_sub = zeros(nROIs, nGrayVox, 'single'); % Pearson r values

FZ_sub = zeros(nROIs, nGrayVox, 'single'); % Fisher z (atanh)

SZ_sub = zeros(nROIs, nGrayVox, 'single'); % Within-ROI standardized z

for j = 1:nROIs

seedIdx = customROI_vox{j};

if isempty(seedIdx)

% If ROI contains no voxels, fill with NaN to mark invalid seed

r_vox = NaN(nGrayVox,1);

else

% Compute seed timeseries as the mean across ROI voxels.

% VxT rows are voxels, columns are timepoints -> take mean

seedTS = mean(VxT(seedIdx, :), 1)'; % [T x 1]

if all(isnan(seedTS)) || std(seedTS) == 0

% If seed timeseries is constant or NaN, correlations undefined

r_vox = NaN(nGrayVox,1);

else

% Compute Pearson correlation between seedTS and each gray voxel

% corr expects inputs as columns: we pass grayVxT' versus seedTS

r_vox = corr(grayVxT', seedTS); % [nGrayVox x 1]

end

end

% Numerical safeguards before Fisher transform:

% clip correlations to avoid atanh divergence at 卤1

r_vox = min(max(r_vox, -0.99), 0.99);

% Fisher z-transform

z_fz = atanh(r_vox);

% Within-ROI standardization: compute mean and std of r across

% gray voxels and z-score. Handle zero or NaN sigma by assigning

% zeros to maintain consistency for downstream procedures.

mu = nanmean(r_vox);

sigma = nanstd(r_vox);

if sigma == 0 || isnan(sigma)

z_sz = zeros(size(r_vox), 'like', r_vox); % avoid NaN-filled matrices

else

z_sz = (r_vox - mu) ./ sigma;

end

% Store results in preallocated matrices (ensure correct shape)

FC_sub(j, :) = reshape(single(r_vox), 1, []); % original r

FZ_sub(j, :) = reshape(single(z_fz), 1, []); % Fisher z

SZ_sub(j, :) = reshape(single(z_sz), 1, []); % within-ROI z

% Send progress update (prints a dot)

send(dq, 1);

end

% Save the three subject-level matrices to disk using -v7.3

outFileFC = fullfile(outFC, sprintf('%s_ROI_Gray_FC.mat', subjName));

outFileFZ = fullfile(outFZ, sprintf('%s_ROI_Gray_FZ.mat', subjName));

outFileSZ = fullfile(outSZ, sprintf('%s_ROI_Gray_SZ.mat', subjName));

save_subj_mats(outFileFC, outFileFZ, outFileSZ, FC_sub, FZ_sub, SZ_sub, niiList, grayMatter_vox);

catch ME

% Catch and report subject-level errors without terminating the loop.

fprintf('Error %s: %s\n', subjName, ME.message);

end

end

% Save subject list and ROI list to the output directory to facilitate

% subsequent group-level aggregation or quality control.

save(fullfile(outDir, 'subjects_list_and_rois.mat'), 'subjNames', 'niiList', 'grayMatter_vox', '-v7.3');

disp('ROI-to-GrayMatter functional connectivity computation completed. Each subject saved as three files (FC, FZ, SZ).');

%% ------------------------------------------------------------------------

% Subfunction: save_subj_mats

% A small wrapper to centralize saving of the three subject-level files.

% This helper is separated to ensure parfor transparency and to keep the

% parfor body concise.

% -------------------------------------------------------------------------

function save_subj_mats(outFileFC, outFileFZ, outFileSZ, FC_sub, FZ_sub, SZ_sub, niiList, grayMatter_vox)

% Save subject-level matrices with metadata in -v7.3 format to support

% large arrays. The saved variables include:

% - FC_sub : [nROIs x nGrayVox] Pearson r

% - FZ_sub : [nROIs x nGrayVox] Fisher z

% - SZ_sub : [nROIs x nGrayVox] Within-ROI standardized z

% - niiList : structure list of ROI filenames used to index rows

% - grayMatter_vox : linear indices of gray voxels corresponding to columns

save(outFileFC, 'FC_sub', 'niiList', 'grayMatter_vox', '-v7.3');

save(outFileFZ, 'FZ_sub', 'niiList', 'grayMatter_vox', '-v7.3');

save(outFileSZ, 'SZ_sub', 'niiList', 'grayMatter_vox', '-v7.3');

end

**Supplementary Method 4. Code of Dominance Analysis (matlab).**

function [AverageContributionbypredictor, DominanceMat, HR2] = function_DominanceAnalysis(Y, X)

% DominanceMat.DominanceMat_4Complete: i.e.: (i, j) = 1 -> i has Complete Dominance on j

% DominanceMat_4Conditional

% DominanceMat_4Genetal

% X = rand(255, 3);

% Y = rand(255, 1);

Y = zscore(Y, 0, 1);

X = zscore(X, 0, 1);

P = size(X, 2);

P = 1:P;

P = P(:);

H = {};

for i = 1:length(P)

% H1 = nchoosek(1:P, i);

% mat2cell(H1, [])

H = [H; nchoosek(1:length(P), i)];

end

HR2 = cell(size(H, 1), 3);

for ri = 1:size(H, 1)

TemH = H{ri, 1};

temHR2 = nan(size(TemH, 1), 1);

temHR2D = nan(size(TemH, 1), length(P));

for rj = 1:size(TemH, 1)

% parfor rj = 1:size(TemH, 1)

temHR2D_O = nan(1, length(P));

Indx = TemH(rj, :);

TemX = X(:, Indx);

mdl = fitlm(TemX, Y);

TemR2Ref = mdl.Rsquared.Ordinary;

% TemR2Ref = mdl.Rsquared.Adjusted;

temHR2(rj, 1) = TemR2Ref;

TemP = setxor(P, Indx);

if isempty(TemP)

continue

end

for ti = 1:size(TemP, 1)

TemIndx = TemP(ti);

TemX = X(:, [Indx(:); TemIndx]);

mdl = fitlm(TemX, Y);

TemR2Opti = mdl.Rsquared.Ordinary;

% TemR2Opti = mdl.Rsquared.Adjusted;

temHR2D_O(1, TemIndx) = TemR2Opti-TemR2Ref;

end

temHR2D(rj, :) = temHR2D_O;

end

HR2{ri, 1} = H{ri, 1};

HR2{ri, 2} = temHR2;

HR2{ri, 3} = temHR2D;

end

temHR2D = nan(1, length(P));

for si = 1:length(P)

Indx = P(si, :);

TemX = X(:, Indx);

mdl = fitlm(TemX, Y);

TemR2Ref = mdl.Rsquared.Ordinary;

% TemR2Ref = mdl.Rsquared.Adjusted;

temHR2D(Indx) = TemR2Ref;

end

HR2 = [{[], 0, temHR2D}; HR2];

dR2A = zeros(size(HR2, 1), length(P));

for di = 1:size(HR2, 1)

dR2A(di, :) = mean(HR2{di, 3}, 1, 'omitnan');

end

AverageContributionbypredictor = sum(dR2A, 1, 'omitnan')./(size(HR2, 1)-1);

%%

DominanceMat_4Complete = ones(length(P), length(P));

for ri = 1:size(HR2, 1)

TemH = HR2{ri, 3};

for si = 1:size(TemH, 1)

% TemH = [TemH, nan];

[~, I] = sort(TemH(si, :), 'descend');

I = I(:);

Tag_IsNaNb = isnan(TemH(si, :));

Tag_IsNaN = find(isnan(TemH(si, :)));

Tag_IsDel = sum(I == Tag_IsNaN, 2);

Tag_IsDel = logical(Tag_IsDel);

I(Tag_IsDel) = [];

if isempty(I)

continue

end

TemDominanceMat = zeros(size(DominanceMat_4Complete));

for Ii = 1:length(I)-1

CI = I(Ii);

TI = I(Ii+1:end);

TemDominanceMat(CI, TI) = 1;

end

DominanceMat_4Complete(~Tag_IsNaNb, ~Tag_IsNaNb) = ...

DominanceMat_4Complete(~Tag_IsNaNb, ~Tag_IsNaNb).*TemDominanceMat(~Tag_IsNaNb, ~Tag_IsNaNb);

end

end

DominanceMat_4Conditional = ones(length(P), length(P));

for ri = 1:size(dR2A, 1)

TemH = dR2A(ri, :);

Tag_IsNaN = find(isnan(TemH));

[~, I] = sort(TemH, 'descend');

I = I(:);

Tag_IsDel = sum(I == Tag_IsNaN, 2);

Tag_IsDel = logical(Tag_IsDel);

I(Tag_IsDel) = [];

if isempty(I)

continue

end

TemDominanceMat = zeros(size(DominanceMat_4Conditional));

for Ii = 1:length(I)-1

CI = I(Ii);

TI = I(Ii+1:end);

TemDominanceMat(CI, TI) = 1;

end

DominanceMat_4Conditional = DominanceMat_4Conditional.*TemDominanceMat;

end

DominanceMat_4Genetal = ones(length(P), length(P));

TemH = AverageContributionbypredictor;

Tag_IsNaN = find(isnan(TemH));

[~, I] = sort(TemH, 'descend');

I = I(:);

Tag_IsDel = sum(I == Tag_IsNaN, 2);

Tag_IsDel = logical(Tag_IsDel);

I(Tag_IsDel) = [];

TemDominanceMat = zeros(size(DominanceMat_4Genetal));

for Ii = 1:length(I)-1

CI = I(Ii);

TI = I(Ii+1:end);

TemDominanceMat(CI, TI) = 1;

end

DominanceMat_4Genetal = DominanceMat_4Genetal.*TemDominanceMat;

DominanceMat.DominanceMat_4Complete = DominanceMat_4Complete;

DominanceMat.DominanceMat_4Conditional = DominanceMat_4Conditional;

DominanceMat.DominanceMat_4Genetal = DominanceMat_4Genetal;

end

**Supplementary Figure 1. LOI Analysis.**


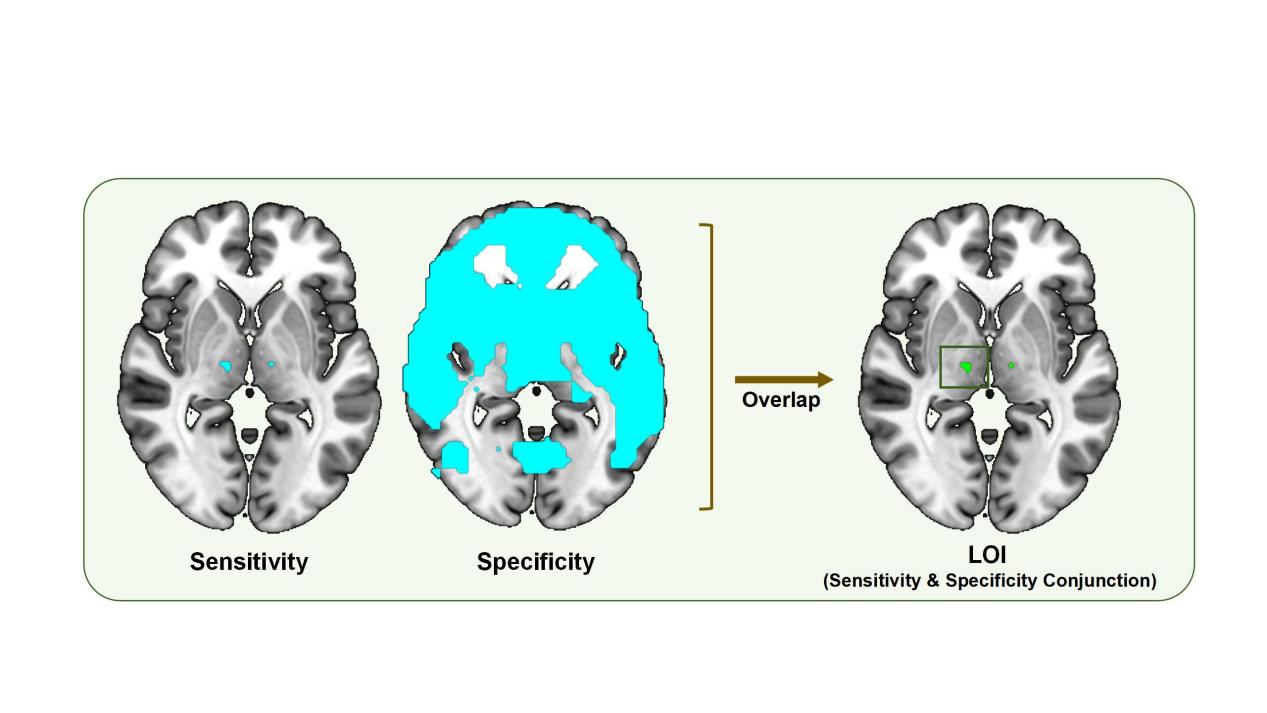


Note: All lesions are depicted from the original anatomical images. LOI: Lesion of Interest.

**Supplementary Table 1. Correlation Analysis of Cognitive Scores (controlling lesion volume).**

| MoCA | Total | Execution | Language | Attention＆Caculation | Memory | Orientation |
| --- | --- | --- | --- | --- | --- | --- |
| FC  (THA.L-SFGmed.L) | *r* = 0.245  *p* = 0.074 | *r* = 0.133  *p* = 0.336 | *r* = 0.182  *p* = 0.188 | *r* = 0.254  *p* = 0.064 | *r* = 0.109  *p* = 0.434 | ***r* = 0.346**  ***p* = 0.010** |
| MMSE | Total | Execution | Language | Attention＆Caculation | Memory | Orientation |
| FC  (THA.L-SFGmed.L) | ***r* = 0.393**  ***p* = 0.003** | ***r* = 0.353**  ***p* = 0.009** | ***r* = 0.312**  ***p* = 0.022** | *r* = 0.213  *p* = 0.123 | ***r* = 0.334**  ***p* = 0.014** | ***r* = 0.369**  ***p* = 0.006** |

Note: MoCA: Montreal Cognitive Assessment; MMSE: Mini-Mental State Examination; FC: Functional Connectivity; THA: Thalamus; SFGmed: SFGmed: Medial Superior Frontal Gyrus.
